# *Mycobacterium bovis* Induces Endoplasmic Reticulum Stress Mediated-Apoptosis by Activating IRF3 in a Murine Macrophage Cell Line

**DOI:** 10.3389/fcimb.2016.00182

**Published:** 2016-12-12

**Authors:** Yongyong Cui, Deming Zhao, Srinand Sreevatsan, Chunfa Liu, Wei Yang, Zhiqi Song, Lifeng Yang, Paul Barrow, Xiangmei Zhou

**Affiliations:** ^1^State Key Laboratories for Agrobiotechnology, Key Lab of Animal Epidemiology and Zoonosis, Ministry of Agriculture, National Animal Transmissible Spongiform Encephalopathy Laboratory, College of Veterinary Medicine, China Agricultural UniversityBeijing, China; ^2^Veterinary Population Medicine Department, College of Veterinary Medicine, University of MinnesotaSt. Paul, MN, USA; ^3^School of Veterinary Medicine, University of NottinghamSutton Bonington, UK

**Keywords:** mycobacterium, *M. bovis*, *ER stress*, *IRF3*, *apoptosis*

## Abstract

Mycobacterium bovis (*M. bovis*) is highly adapted to macrophages and has developed multiple mechanisms to resist intracellular assaults. However, the host cells in turn deploy a multipronged defense mechanism to control bacterial infection. Endoplasmic reticulum (ER) stress-mediated apoptosis is one such primary defense mechanism. However, the role of interferon regulatory factor 3 (IRF3) between ER stress and apoptosis during *M. bovis* infection is unknown. Here, we demonstrate that *M. bovis* effectively induced apoptosis in murine macrophages. Caspase-12, caspase-9, and caspase-3 were activated over a 48 h infection period. The splicing of XBP-1 mRNA and the level of phosphorylation of eIF2α, indicators of ER stress, significantly increased at early time points after *M. bovis* infection. The expansion of the ER compartment, a morphological hallmark of ER stress, was observed at 6 h. Pre-treatment of Raw 264.7 cells with 4-PBA (an ER stress-inhibitor) reduced the activation of the ER stress indicators, caspase activation and its downstream poly (ADP-ribose) polymerase (PARP) cleavage, phosphorylation of TBK1 and IRF3 and cytoplasmic co-localization of STING and TBK1. *M. bovis* infection led to the interaction of activated IRF3 and cytoplasmic Bax leading to mitochondrial damage. Role of IRF3 in apoptosis was further confirmed by blocking this molecule with BX-795 that showed significant reduction expression of caspase-8 and caspase-3. Intracellular survival of *M. bovis* increased in response to 4-PBA and BX-795. These findings indicate that STING-TBK1-IRF3 pathway mediates a crosstalk between ER stress and apoptosis during *M. bovis* infection, which can effectively control intracellular bacteria.

## Introduction

It is more than a hundred years since *Mycobacterium tuberculosis* (Mtb) was first isolated by Robert Koch (Cui et al., 2016). Tuberculosis (TB) remains one of the most serious global diseases affecting humans and animals (WHO, 2014). *Mycobacterium bovis* (*M. bovis*), a species belonging to *Mycobacterium tuberculosis* Complex, is the main cause of TB in cattle, deer and other mammals and it also can infect people by drinking or eating contaminated, unpasteurized dairy products or close contact with infected animals (Arap et al., 2004; Waters and Palmer, 2015). Macrophages are the major cell type infected by Mtb and *M. bovis*. Consequently, host innate immune mechanisms have co-evolved to better counter mycobacterial infections (Aldwell et al., 1996). Host macrophages also induce an apoptotic signal to control bacterial infection (Rodrigues et al., 2013; Srinivasan et al., 2014). Understanding the mechanism of apoptosis induced by *M. bovis* infection will lead to better delineate host immune responses that could be exploited to control infection.

Endoplasmic reticulum (ER) is not only the major site of folding and transportation of proteins after synthesis, but also the major site of storage of intracellular Ca^2+^ and synthesis of cholesterol, steroids, and lipids. Recent studies have demonstrated that infection of macrophages by the virulent H37Rv and attenuated H37Ra strains results in increases in the amount of rough- (RER) and smooth- (SER) endoplasmic reticulum respectively (Saquib et al., 2015). And mycobacterial infection results in loss of Ca^2+^ from the ER and an increase in the intracellular redox state which results in accumulation of unfolded or misfolded proteins in the ER resulting in ER stress, characterized by an expansion in the ER compartment (Choi et al., 2010; Verfaillie et al., 2012; Lim et al., 2013, 2015). The host cell responds with unfolded protein response (UPR) which suspends the synthesis of new proteins and thereby reduces accumulation of unfolded or misfolded proteins in the ER to restore normal physiological function (Ron and Walter, 2007). Prolonged and uncontrolled ER stress can lead to activation of a triad of signaling pathways moving the cell toward apoptosis (Tabas and Ron, 2011; Hetz, 2012). Inositol-requiring enzyme 1 (IRE1), protein kinase RNA (PKR)-like ER kinase (PERK), and activating transcription factor 6 (ATF6) are mainly three pathways that participate in ER stress (Rasheva and Domingos, 2009; Sano and Reed, 2013). However, it is unknown whether ER stress is also involved in *M. bovis* infection and the mechanism of macrophages apoptosis.

It is well-known that stimulator of interferon genes (STING) is located in the outer membrane of the ER. Recent research has documented that ER stress causes the translocation of STING into the cytoplasm, where STING recruits TANK-binding kinase 1 (TBK1) to activate interferon regulatory factor 3 (IRF3) (Liu et al., 2012; Petrasek et al., 2013). Activated IRF3 likely causes the release of cytochrome c by a variety of mechanisms, leading to the damage of mitochondria and eventually, cell death (Chattopadhyay et al., 2010; Vince and Tschopp, 2010).

Here we show that ER stress of macrophages leads to apoptosis in a caspase-dependent manner during *M. bovis* infection. And we demonstrate that ER stress-induced apoptosis is mediated by the activation of IRF3, which requires STING and TBK1. Both mechanisms effectively control intracellular *M. bovis* killing. To our knowledge, this is the first report to investigate ER stress in *M. bovis* infection and it's association with IRF3 release leading to apoptosis.

## Materials and methods

### Reagents and antibodies

Rabbit monoclonal anti-phospho-eIF2α (Ser51) antibody, rabbit monoclonal anti-phospho-IRF-3 (Ser396) antibody were acquired from Cell Signaling Technology (Danvers, MA, USA). Rabbit monoclonal anti-TBK1 antibody was purchased from Abcam (Cambridge, UK). Rabbit polyclonal anti-STING antibody, rabbit polyclonal anti-PARP antibody, and rabbit polyclonal anti-tubulin antibody were obtained from Santa Cruz Biotechnology (Santa Cruz, CA, USA). Rabbit polyclonal anti-caspase-3 (p17 specific) antibody, rabbit polyclonal anti-caspase-8 antibody, rabbit polyclonal anti-caspase-9 antibody, rabbit polyclonal anti-caspase-12 antibody, rabbit polyclonal anti-BAX antibody, and rabbit polyclonal anti-cytochrome c antibody were purchased from Proteintech (Wuhan, China). Rabbit polyclonal anti-VDAC1 was acquired from Sangon Biotech (Shanghai, China). Peroxidase-Conjugated Affinipure goat anti-rabbit IgG(H+L) (ZB- 5301) and goat anti-mouse IgG(H+L) (ZB- 5305), Alexa Fluor 488-conjugated Affinipure goat anti-rabbit IgG(H+L) (ZB- 0511) were purchased from ZSGB Biotechnology (Beijing, China). Donkey anti-goat IgG/Alexa Fluor 647 was purchased from Biosynthesis Biotechnology (Beijing, China). Tunicamycin was purchased from Fermentek Ltd (Jerusalem, Israel). BX-795 was purchased from Selleckchem (Houston, TX, USA).

### Cell culture

Murine macrophage Raw 264.7 cell line was obtained from China Infrastructure of Cell Line Resources (Beijing, China), and was maintained in Dulbecco's modified Eagle's medium (HyClone, South Logan, Utah, USA) supplemented with 10% fetal bovine serum, 1% L-glutamine (Gibco, Grand Island, NY, USA), penicillin (10,000 units/ml), streptomycin (10,000 μg/ml) (HyClone, South Logan, Utah, USA) at 37°C with 5% CO_2_.

### M. *bovis* culture

*M. bovis* Beijing strain was obtained from China Institute of Veterinary Drug Control (CVCC, China) and was grown in Middlebrook 7H9 liquid medium (BD Biosciences, NY, USA) with 10% ADC (albumin, dextrose, catalase), and 0.05% Tween-80 and grown to mid-log phase for 1 week at 37°C. Aliquots were frozen at −80°C until used.

### *M. bovis* infection and colony-forming unit assay

Macrophages were infected *in vitro* with *M. bovis* at an MOI of 10 and incubated for 3 h at 37°C with 5% CO_2_. After allowing 3 h for phagocytosis, the cells were washed three times with warm PBS to remove extracellular bacteria and then incubated with fresh medium without antibiotics for an additional time. They were then lysed in autoclaved distilled water to allow collection of intracellular bacteria. The lysates were plated separately on Middlebrook 7H11 agar plates and incubated at 37°C with 5% CO_2_ for 3 weeks. Colony counts were performed in triplicate.

### Apoptosis analysis

Apoptotic cells were assessed using an Annexin V/propidium iodide (AV/PI) staining kit according to the manufacturer's instructions (KeyGENBioTECH, Nanjing, China). Binding of Annexin V and PI was analyzed by BD FACSCalibur flow cytometer (BD Biosciences, San Jose, CA, USA) with FlowJo software (Tree Star, Ashland, OR, USA).

### Transmission electron microscopy

Cells were infected and then washed three times with PBS, trypsinized, fixed in ice-cold 5% glutaraldehyde in 0.1 M sodium cacodylate buffer (pH 7.4) at 4°C for 15 min, and then centrifuged. Cell pellets were fixed for 4 h. After a complete rinse with sodium cacodylate buffer, the cell pellet was further fixed in 1% OsO4 in 0.1 M sodium cacodylate buffer on ice for 1 h and dehydrated with acetone. The cell pellet was embedded in EM-bed 812 resin and polymerized at 60°C for 48 h. Ultrathin sections (70 nm) were obtained on a Leica Ultracut UCT ultramicrotome (Vienna, Austria) and counterstained with uranyl acetate and lead citrate before observation under a fluorescence microscope (Olympus Fluoview, Japan).

### RT-PCR analysis

Total RNA was extracted using the EASYspin Plus RNA Extraction Kit (Aidlab, Beijing, China). Then total RNA was reverse-transcribed to cDNA using the Maxima First Strand cDNA synthesis kit (Thermo Scientific, Waltham, MA, USA). For determination of XBP-1 splicing, the cDNA product was subjected to 35 cycles of PCR using the forward primer 5′-AAACAGAGTAGCAGCTCAGACTGC-3′ and the reverse primer 5′-TCCTTCTGGGTAGACCTCaTGGGAG-3′ which are specific for mouse XBP-1. β-actin (forward: CCTTCTGACCCATTCCCACC; reverse: GCTTCTTTGCAGCTCCTTCG) was used as an housekeeping control. Products were separated by electrophoresis through a 3% agarose gel.

### Immunofluorescence microscopy

For protein localization analysis, Raw 264.7 cells grown on cover slips were washed twice with PBS, fixed by Immunol Staining Fix Solution, blocked 1 h at room temperature by Immunol Staining Blocking Buffer (Beyotime Biotechnology, Shanghai, China) and then incubated overnight at 4°C with the appropriate primary and secondary antibodies. The nuclei were stained with DAPI. Raw 264.7 cells were labeled using anti-phospho-IRF3 antibody, anti-STING antibody, anti-TBK1 antibody, and fluorescein isothiocyanate (FITC)-conjugated donkey anti-goat, Alexa Fluor-conjugated goat anti-rabbit and FITC-conjugated goat anti-rabbit antibodies as the primary and secondary antibodies respectively. Macrophages were finally mounted with glycerin buffer and examined immediately with an OLYMPUS microscope. All experiments were performed on three independent occasions in triplicate at each time.

### Western blot and co-immunoprecipitations

Cellular proteins were extracted with lysis buffer and total protein concentrations were determined with the Bradford assay and 30 mg of protein was separated with SDS-PAGE, followed by electrotransfer to a Immobilon-P Membrane (Millipore, MA, USA). The blots were probed with primary antibodies and secondary antibodies at optimized concentrations. Enhanced chemiluminescence was used as WB detection system. Image J (NIH, Washington, C, USA) was used for quantification of immunoblots. For immunoprecipitation, cell lysates were pre-cleared using protein A/G agarose (Santa Cruz, CA, USA) beads at 4°C for 30 min on a rocker. The beads were removed by centrifugation at 13,200 rpm at 4°C for 1 min and transferred the supernatant to a fresh centrifuge tube. Then protein A/G agarose/sepharose slurry was incubated with anti-IRF3 antibody overnight at 4°C. Beads were washed 3–4 times with RIPA or NP40 buffer. The agarose/sepharose beats were resuspended in the sample buffer and were boiled for 5 min. The beats were then spinned down and the supernatants were used for SDS-PAGE and western blot analysis.

### Isolation of mitochondria and cytoplasm

Isolation of mitochondria and cytoplasm from Raw 264.7 whole cell lysates was performed with the Subcellular Structure Mitochondrial Isolation Kit and the Subcellular Structure Cytoplasmic and Nuclear Isolation Kit respectively according to manufacturer's protocol (Boster, Wuhan, China).

### Mitochondrial transmembrane potential (ΔΨ_m_) assay

Mitochondrial transmembrane potential was assessed using the cationic fluorescent indicator JC-1 (Molecular Probes, Eugene, OR, USA), which aggregates in intact mitochondria (red fluorescence) with normal ΔΨm, but remains in the monomeric form in the cytoplasm (green fluorescence) of cells with disrupted mitochondrial membrane. Raw 264.7 cells were incubated in DMEM medium containing 10 μM JC-1 at 37°C for 15 min, washed with PBS, and then transferred to a clear 24-well plate. JC-1 aggregate fluorescent emission was measured at 583 nm with an excitation wavelength of 526 nm; and JC-1 monomer fluorescence intensity was measured with excitation and emission wavelengths at 525 and 530 nm. Finally, the cells were mounted with the DakoCytomation fluorescent medium and visualized *via* fluorescence microscopy (Olympus Fluoview, Japan).

### Statistical analysis

All assays were performed on three separate occasions in triplicate at each time. Results are expressed as means ± *S.D*. All comparisons of data were made using one-way ANOVA followed by *post-hoc* Tukey's test or Student's *t*-test. SPSS software (version 13.0: SPSS Inc., Chicago, IL, USA), GraphPad Prism 5 software (La Jolla, CA, USA) and Image J (National Institutes of Health, USA) were used, and *P* < 0.05 was considered significant.

## Results

### *M. bovis* infection induces apoptosis

**Figure 1:**
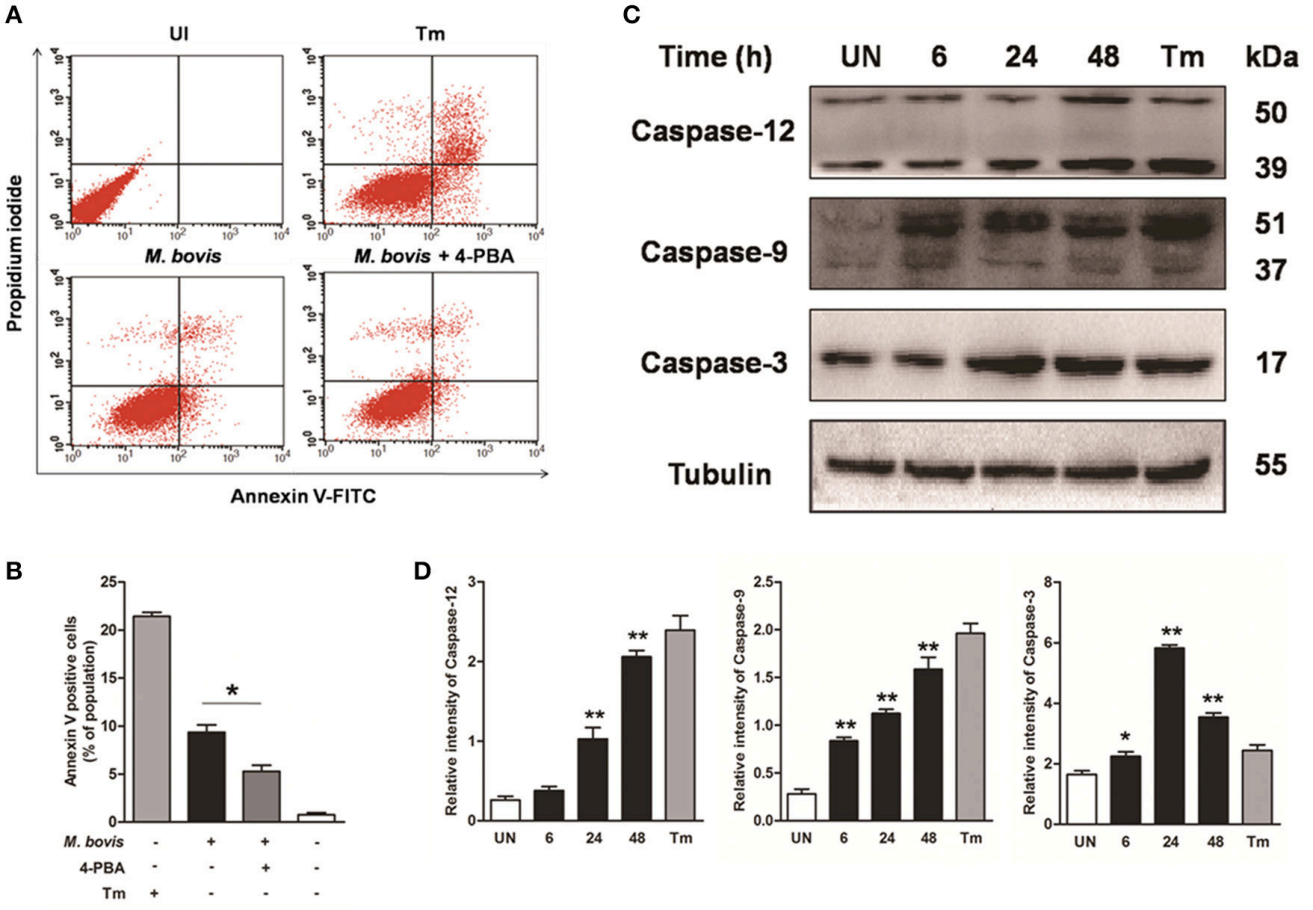
*****M. bovis*** infection induces apoptosis and caspase activation**. **(A)** Raw 264.7 cells were screened for induction of apoptosis using Annexin V/PI staining after 24 h infection with *M. bovis* at an MOI of 10. Tunicamycin (5 μg/ml) was used as a positive control for apoptosis. 4-PBA, an ER stress inhibitor was treated before *M. bovis* infection in Raw 264.7 cells. After washing and Annexin V/PI staining, cells were analyzed by flow cytometry. **(B)** Quantitative analysis of the percentage of apoptotic cells (sum of early and late apoptotic cells) from **(A)**, a statistically significant difference (^*^P < 0.05) is observed between 4-PBA pretreated and non-pretreated groups using the two-tailed *t*-test. **(C)** Raw 264.7 cells were stimulated with *M. bovis* for 24 h and total cell lysates were subjected to Western blot for cleaved caspase-3 (p17 specific), caspase-9, and caspase-12. **(D)** Bands corresponding to each protein were quantified, and the intensities of each protein were normalized to the intensity of tubulin. Data are representative of at least three independent experiments, each performed in triplicate with similar results. The asterisks indicate statistically significant differences compared with untreated cells (^*^P < 0.05, ^**^P < 0.01).

Apoptosis plays an important role in host defense against mycobacterial infection (Xu et al., 2014; Liu et al., 2015). We first investigated if intracellular *M. bovis* could induce apoptosis in macrophages. Initial evaluation of MOI to efficiency of phagocytosis in the RAW 264.7 macrophages showed that 98% of the cells were infected with an MOI of 10 at 24 h. Flow cytometric analysis with Annexin V/propidium iodide (AV/PI) staining was used to quantify cells undergoing apoptosis after 24 h of infection with *M. bovis*. As shown in Figures 1A,B, the percentage of early apoptotic and late apoptotic cells increased from 0.7% in control cells to 9.5% after *M. bovis* infection. Caspases were activated in *M. bovis* infected Raw 264.7 cells. Cleavage of caspase-12 and caspase-9 gradually increased in a time-dependent manner over a 48 h period, while caspase-3 started to decrease by 24 h post infection (pi) (Figures 1C,D). These data indicate that *M. bovis* induces macrophage apoptosis.

### ER stress is induced during *M. bovis* infection

**Figure 2:**
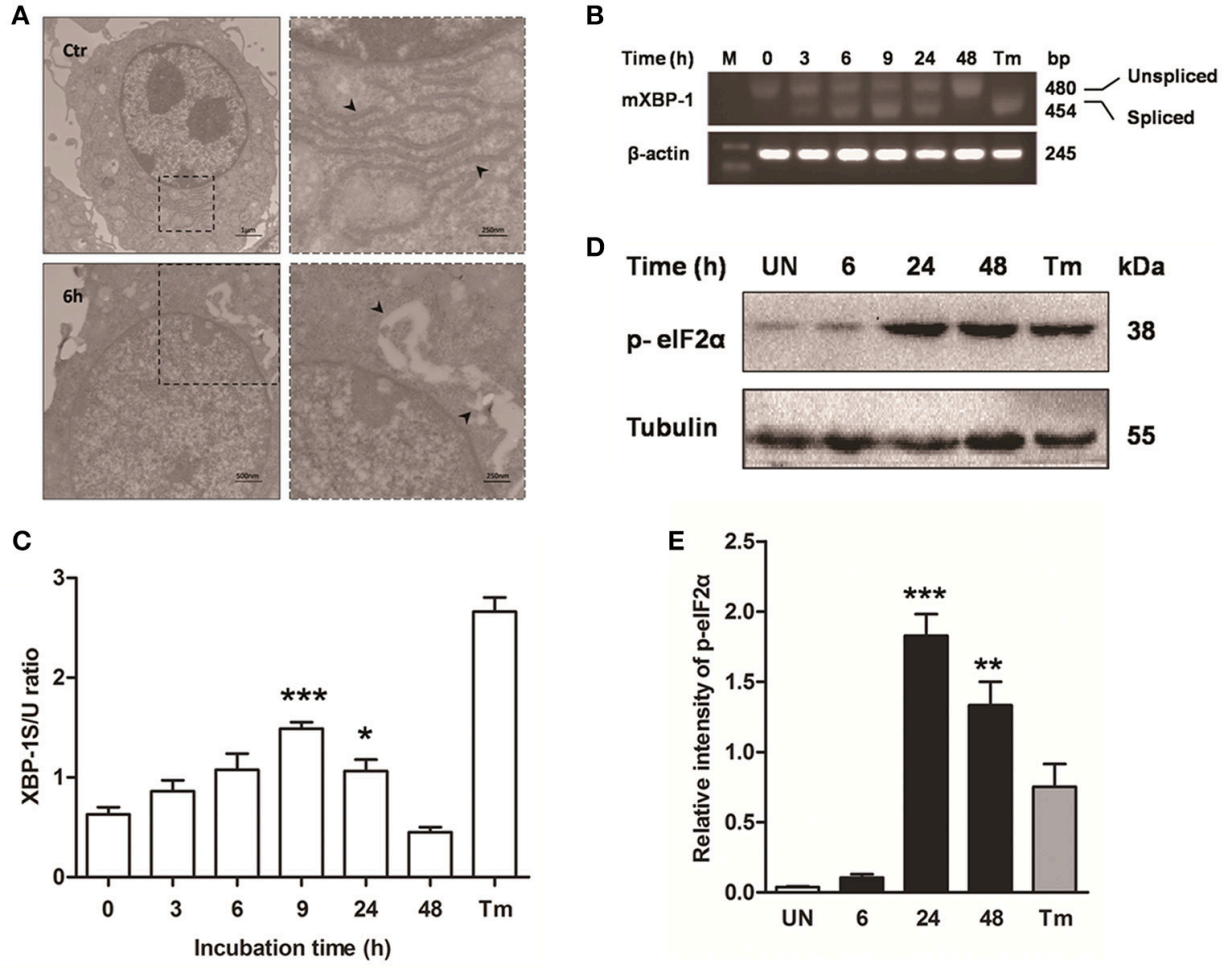
**ER stress is involved in ***M. bovis***-mediated apoptosis**. Raw 264.7 cells were infected with *M. bovis* at an MOI of 10, and then incubated for 0–48 h. **(A)** TEM analysis of Raw 264.7 cells after *M. bovis* infection for 6 h. The perinuclear rough ER regions on the images in the left panel are magnified on the right panels. Arrows indicate ER lamellae before and after infection. **(B)** XBP-1 mRNA splicing was determined by RT-PCR using specific primers that were used to amplify products of unspliced and spliced mRNA. **(C)** The results represent the ratio of spliced XBP-1 to intact (or unspliced) XBP-1 (XBP-1S/U ratio). **(D)** Total cell lysates were subjected to Western blot to identify phosphorylation of eIF2α. **(E)** Bands corresponding to each protein were quantified, and the intensities of each protein were normalized to the intensity of tubulin. Data are representative of at least three independent experiments, each performed in triplicate with similar results. The asterisks indicate significant differences compared with untreated cells (^*^P < 0.05, ^**^P < 0.01, ^***^P < 0.001).

Previous studies have shown that mycobacterium-induced apoptosis is associated with ER stress (Seimon et al., 2010; Sohn et al., 2011). To determine if *M. bovis*-induced apoptosis was also linked to ER stress, morphology of ER was detected by transmission electron microscopy (TEM). We found expansion of the ER compartment within 6 h after *M. bovis* infection, a morphological hallmark of ER stress (Figure 2A). Apoptosis of macrophages was significantly decreased when the chemical chaperone 4-phenyl butyric acid (4-PBA), an ER stress inhibitor, was used to treat cells prior to *M. bovis* infection (Figures 1A,B). ER stress triggers apoptosis mainly through the PERK and IRE1α pathways (Sano and Reed, 2013). To investigate whether these two pathways were activated during *M. bovis* infection in Raw 264.7 cells, we examined the levels of expression of the markers of ER stress. Expression and the splicing of X-box binding protein 1 (XBP-1) mRNA was markedly increased at 9 h pi and then gradually decreased until 48 h pi (Figures 2B,C). Phosphorylation of eukaryotic translation initiation factor 2A (eIF2α) appeared as early as 6 h pi and reached the peak at 24 h pi (Figures 2D,E). These results suggest that *M. bovis* interacts with ER and inducing stress.

### Effect of ER stress-mediated apoptosis on intracellular survival of *M. bovis*

**Figure 3:**
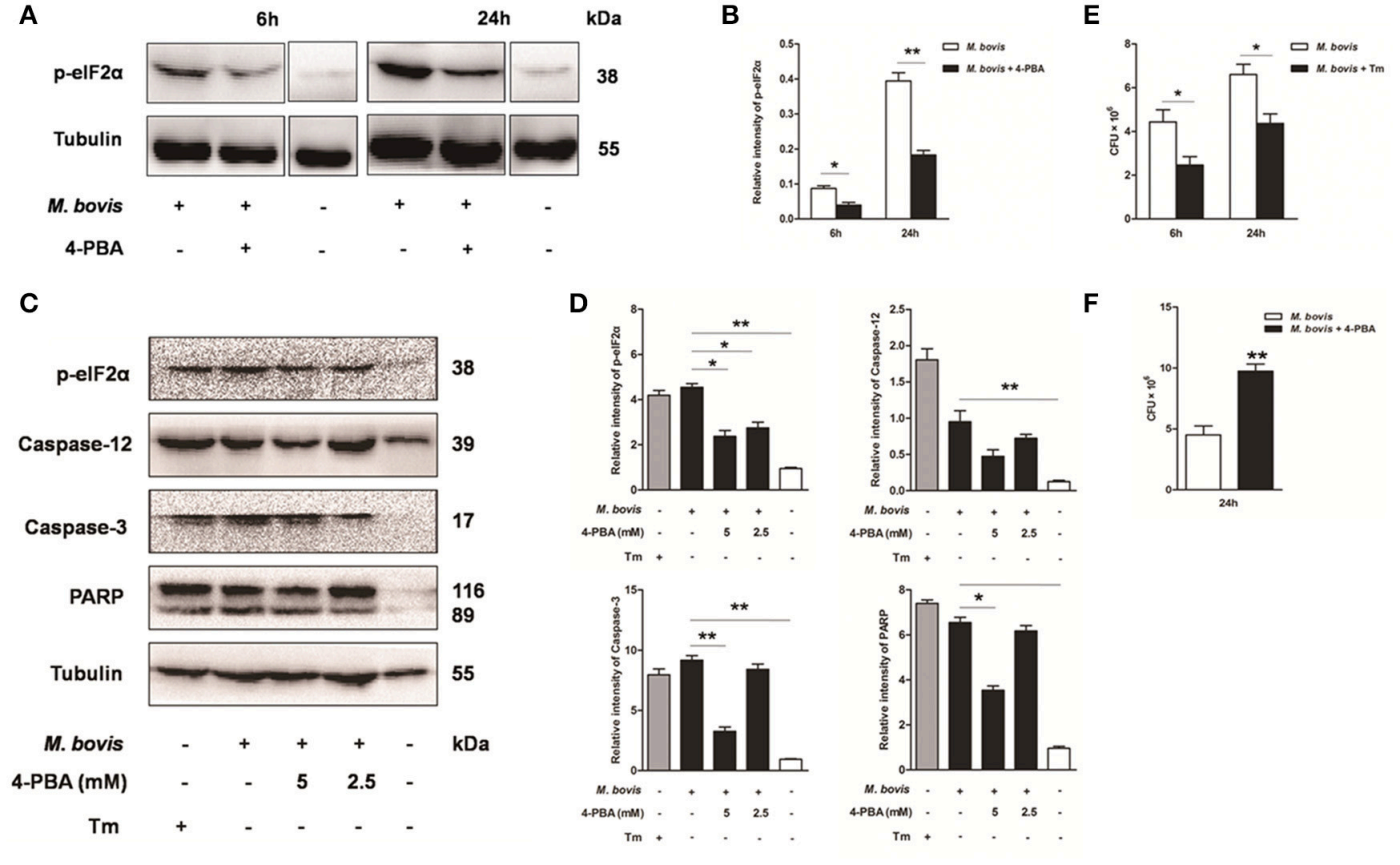
**Effect of ER stress mediated apoptosis on intracellular survival of ***M. bovis*****. **(A–D)** 4-PBA was treated before *M. bovis* infection in Raw 264.7 cells and total cell lysates were subjected to Western blot for phosphorylation of eIF2α, cleaved caspase-12, and caspase-3 (p17 specific) and PARP. Bands corresponding to each protein were quantified, and the intensities of each protein were normalized to the intensity of tubulin. **(E)** Quantification of intracellular survival of *M. bovis* in Raw 264.7 cells pretreated for 3 h with tunicamycin (5 μg/ml). Cells were harvested at 6 and 24 h post infection with *M. bovis* and bacteria number was determined by CFU counting. **(F)** Quantification of intracellular survival of *M. bovis* in Raw 264.7 cells pretreated for 3 h with 4-PBA (5 mM). Cells were harvested at 24 h post infection with *M. bovis* and bacterial numbers were determined. Data are representative of at least three independent experiments, each performed in triplicate with similar results. The asterisks indicate significant differences compared with untreated cells (^*^P < 0.05, ^**^P < 0.01).

Based on above studies, we reasoned that *M. bovis*-induced apoptosis was associated to the ER stress pathways (Lim et al., 2011; Choi et al., 2013). Raw 264.7 cells were treated with 4-PBA before *M. bovis* infection. Phosphorylation of eIF2α was significantly attenuated by the treatment with 5 mM 4-PBA at 6 h and 24 h pi, compared to respective control cells (Figures 3A,B). In response to the treatment with 2.5 mM and 5 mM 4-PBA, phosphorylation of eIF2α and the cleavage of caspase-12 which resides in the ER, caspase-3 and its downstream cleaved poly (ADP-ribose) polymerase (PARP) were attenuated in a dose-dependent manner at 24 h pi (Figures 3C,D). Then we posited that ER stress could influence survival of intracellular bacteria. ER stress agonist tunicamycin (Tm) was used prior to *M. bovis* infection. We found that the total numbers of intracellular bacteria were respectively decreased by 44.4% and 33.8% compared to macrophages without Tm treatment at 6 h and 24 h pi (Figure 3E). We further confirmed that intracellular survival of *M. bovis* was significantly increased by 53.7% in response to 4-PBA at 24 h pi (Figure 3F). These data suggest that *M. bovis*-induced apoptosis is mediated by ER stress, which can effectively control intracellular mycobacteria.

### ER stress results in the phosphorylation and nuclear translocation of IRF3

**Figure 4:**
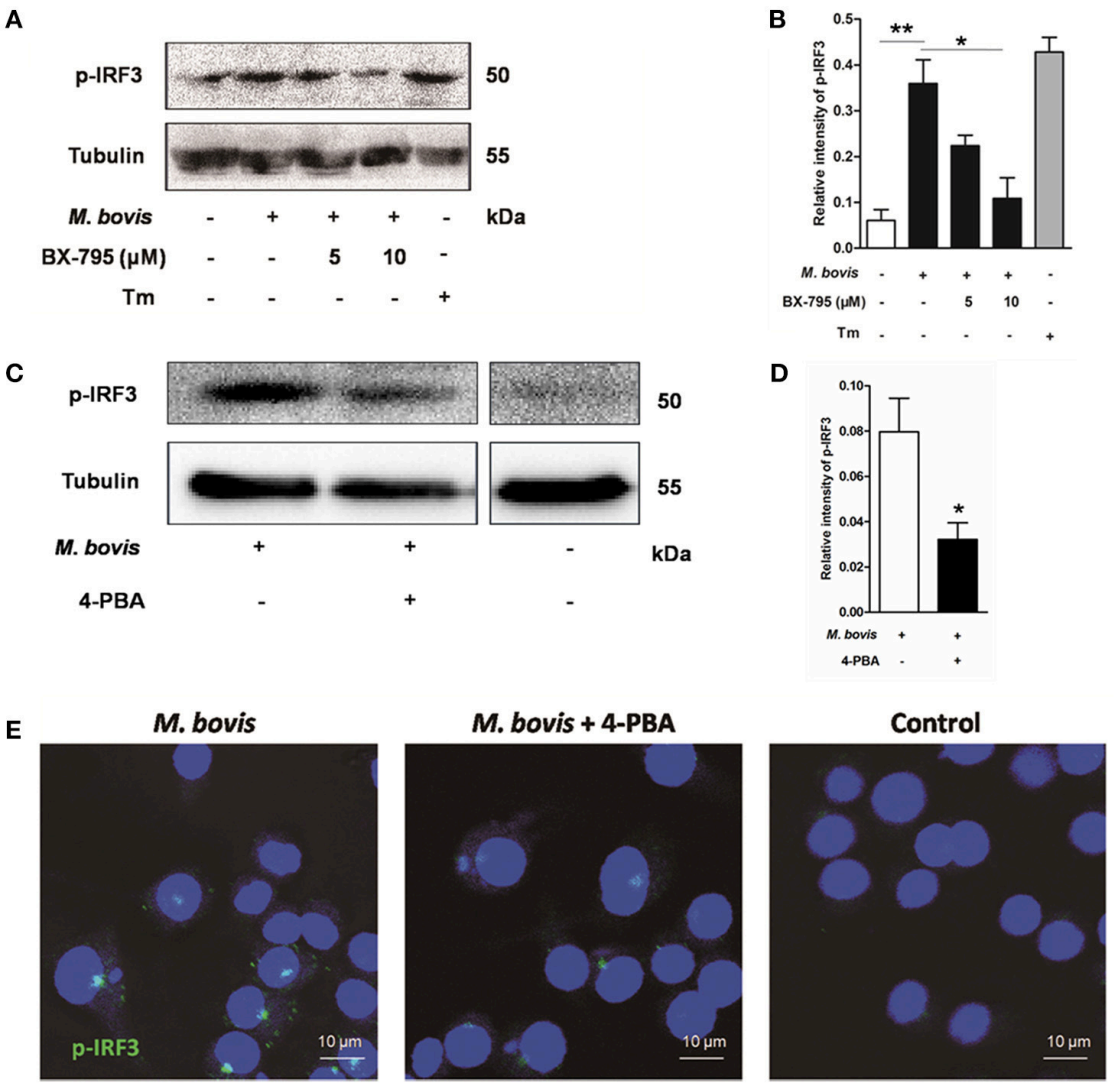
**ER stress results in the phosphorylation and nuclear translocation of IRF3**. **(A–D)** BX-795 and 4-PBA were respectively treated before *M. bovis* infection in Raw 264.7 cells and total cell lysates were subjected to Western blot for phosphorylation of IRF3. Bands corresponding to each protein were quantified, and the intensities of each protein were normalized to the intensity of tubulin. The asterisks indicate significant differences compared with untreated cells (^*^P < 0.05, ^**^P < 0.01). **(E)** 4-PBA was treated before *M. bovis* infection in Raw 264.7 cells. Fixed cells were incubated with anti-IRF3 monoclonal antibody followed by FITC-conjugated goat anti-rabbit antibody as the secondary antibody and visualized by immunofluorescence microscopy. Results are representative of three independent experiments, each performed in triplicate with similar results.

Previous studies have suggested that IRF3, a transcription factor regulating innate immune responses, was involved in ER stress-mediated apoptosis (Liu et al., 2012; Petrasek et al., 2013; Jin et al., 2015). However, the role of IRF3 in *M. bovis* infection is unknown. Thus, the phosphorylation of IRF3 was studied during *M. bovis* infection. Our studies show that phosphorylation of IRF3 ensued *M. bovis* infection at 24 h pi (Figures 4A–D). Nuclear translocation of IRF3 implies that phosphorylation has occurred (Majumdar et al., 2015). We further confirmed that the activation of IRF3 during *M. bovis* infection by immunofluorescence. IRF3 was significantly detected in the nucleus, while no fluorescent clusters were found in uninfected control cells (Figure 4E). Raw 264.7 cells were treated with 5 mM 4-PBA before *M. bovis* infection to verify that phosphorylation of IRF3 was mediated by ER stress. As shown in Figures 4C–E, phosphorylation and nuclear translocation of IRF3 were reduced. These results indicate that ER stress results in the phosphorylation and the nuclear translocation of IRF3 during *M. bovis* infection.

### Activation of IRF3 requires mobilization of STING and TBK1

As an adaptor, STING predominantly resides in the ER. Recent studies have shown that calcium mobilization is involved in ER stress, which causes the translocation of STING and subsequently activates the downstream molecule TBK1 in the cytoplasm. TBK1 is an upstream serine/threonine kinase that plays an essential role in phosphorylating IRF3 (Liu et al., 2012; Tanaka and Chen, 2012; Petrasek et al., 2013). To verify whether the activation of IRF3 was associated with TBK1, Raw 264.7 cells were treated with the potent and specific TBK1 inhibitor BX-795 before *M. bovis* infection. Levels of phosphorylation of IRF3 decreased in a dose-dependent manner in response to 5 and 10 μM BX-795 (Figures 4A,B).

**Figure 5:**
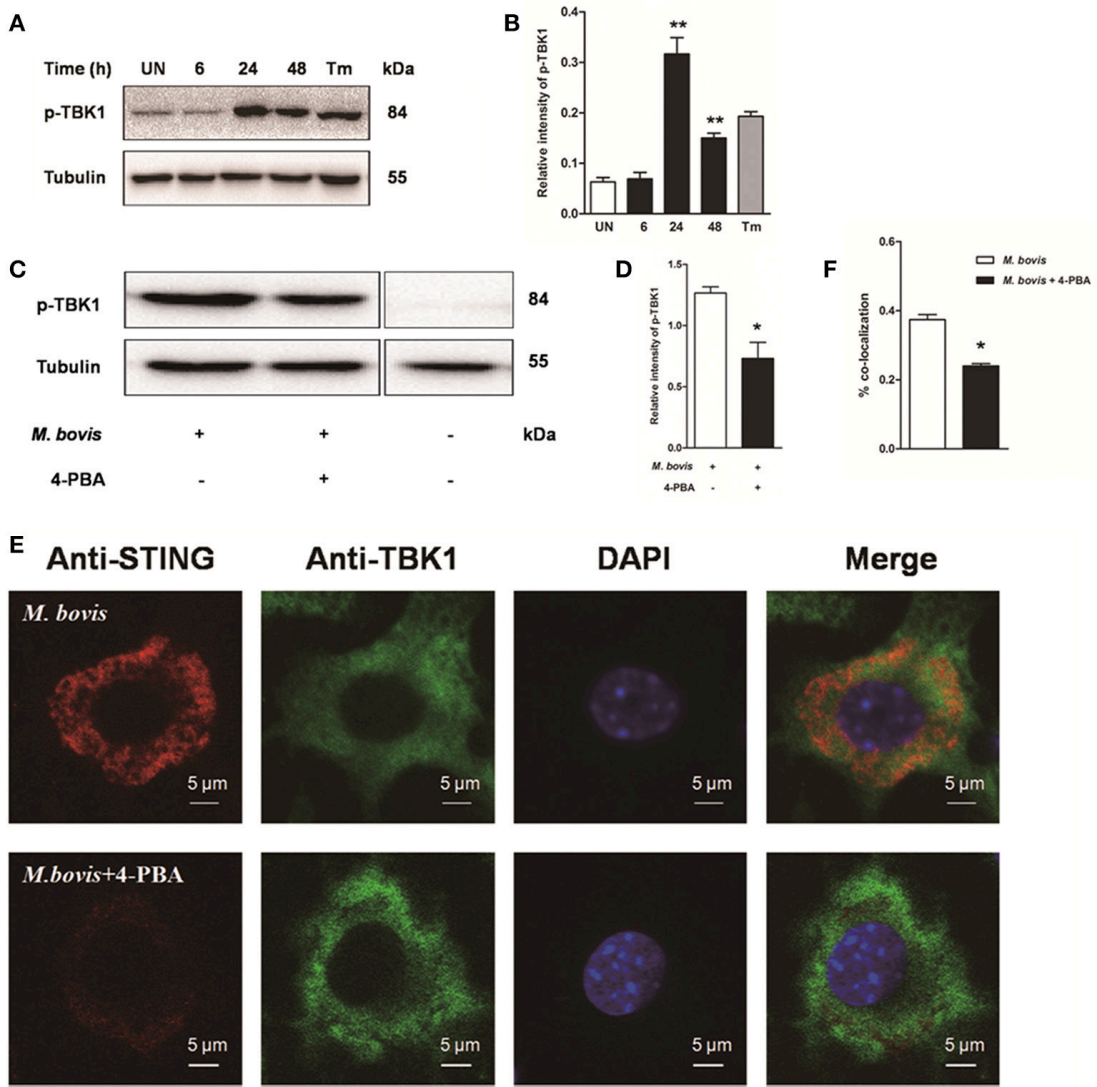
**Activation of IRF3 requires the mobilization of STING and TBK1**. **(A–D)** Raw 264.7 cells were infected with *M. bovis* at an MOI of 10, and then incubated for 0–48 h. Total cell lysates were subjected to Western blot for phosphorylation of TBK1. Bands corresponding to each protein were quantified, and the intensities of each protein were normalized to the intensity of tubulin. **(C,D)** 4-PBA was treated before *M. bovis* infection in Raw 264.7 cells and total cell lysates were subjected to Western blot for phosphorylation of TBK1. **(E,F)** 4-PBA was treated before *M. bovis* infection in Raw 264.7 cells. Fixed cells were incubated with anti-STING antibody and anti-TBK1 antibody followed by fluorescein isothiocyanate (FITC)-conjugated donkey anti-goat (green) or Alexa Fluor-conjugated goat anti-rabbit (red). Results are representative of at least three independent experiments, each performed in triplicate with similar results. The asterisks indicate significant differences compared with untreated cells (^*^P < 0.05, ^**^P < 0.01).

To investigate whether STING-TBK1 pathway was involved in *M. bovis* infection, phosphorylation of TBK1 was studied in a time-course macrophage infection model. We found that phosphorylation of TBK1 increased in 24 h pi and decreased by 48 h pi, but no difference was identified at 6 h pi, compared to uninfected control cells (Figures 5A,B). Next, we studied whether the activation of TBK1 was associated with ER stress. Raw 264.7 cells treated with 4-PBA prior to *M. bovis* infection showed that the phosphorylation of TBK1 was significantly attenuated by the treatment with 4-PBA at 24 h pi (Figures 5C,D).

To determine whether the translocation of STING caused the phosphorylation of TBK1, we interrogated the interaction between STING and TBK1. By immunofluorescence microscopy, a striking co-localization of STING and TBK1 was identified, with association into larger clusters around the nucleus at 24 h pi. But this co-localization was abrogated when cells were pretreated by 4-PBA (Figures 5E,F). Together, these data suggest that the activation of IRF3 requires the mobilization of STING and TBK1, mediated by ER stress during *M. bovis* infection.

### IRF3 is required for mitochondrial damage during *M. bovis* infection

**Figure 6:**
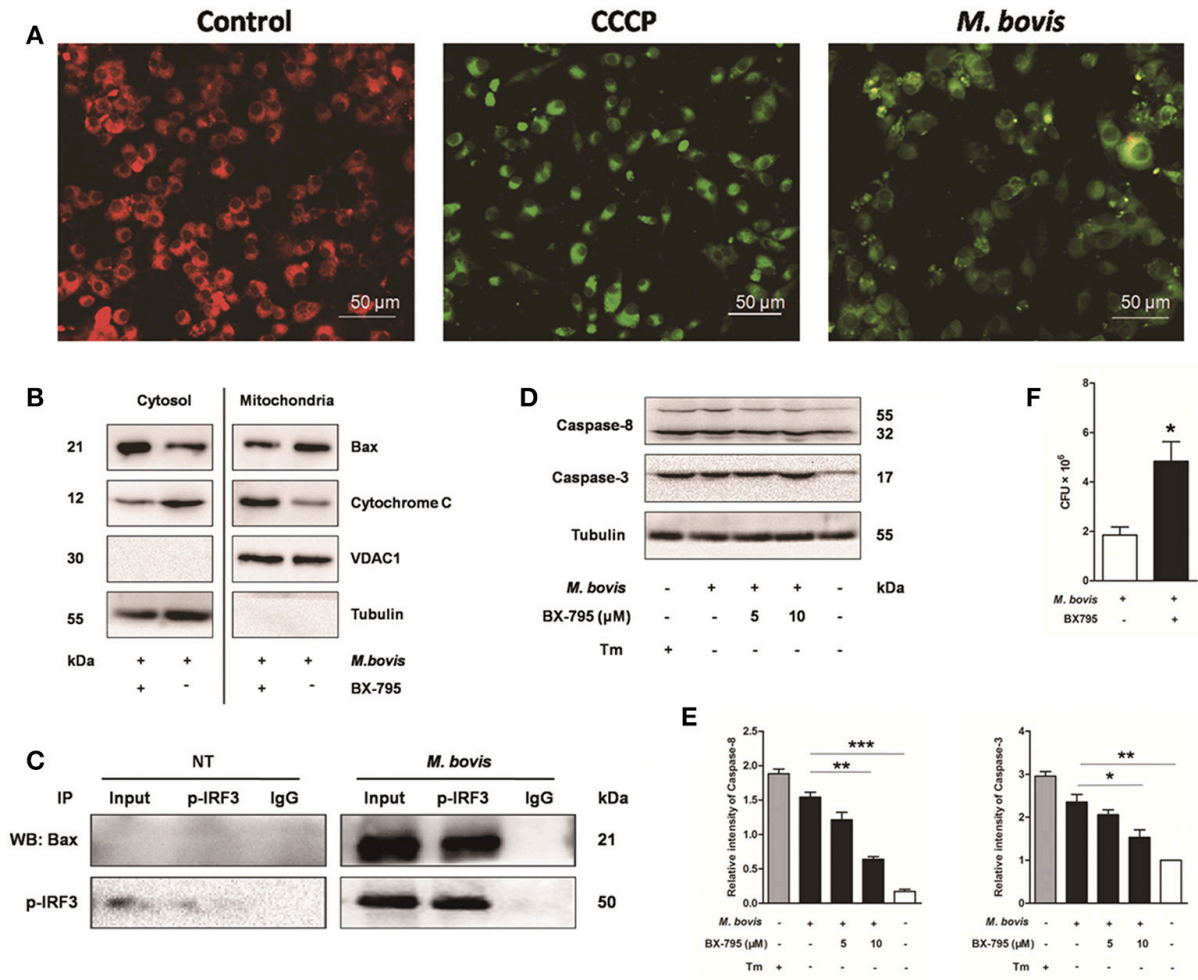
**IRF3 causes the mitochondrial damage and caspase-8 activation**. Raw 264.7 cells were untreated or treated with *M. bovis* at an MOI of 10 for 24 h. **(A)** Mitochondrial transmembrane potential (ΔΨm) is measured using JC-1 as the fluorescent marker. The JC-1-aggregate form, indicating normal ΔΨm, appears red and the monomeric form, indicating low ΔΨm (i.e., disrupted mitochondrial membrane), is green by confocal microscopy. Carbonyl cyanide m-chlorophenylhydrazone (CCCP), as a positive control for the mitochondrial damage. Scale bar = 50 μm. **(B)** Mitochondrial fractions were isolated from whole cell lysates from Raw 264.7 cells. Mitochondrial fractions and supernatant were immunoblotted for the indicated proteins. Left and right parts represent the same membrane. **(C)** Samples were immunoprecipitated with control IgG or anti-phospho-IRF3. Whole-cell lysate (input) or immunoprecipitations were resolved by SDS-PAGE and immunoblotted with anti-phospho-IRF3 or Bax. **(D)** BX-795 was treated before *M. bovis* infection in Raw 264.7 cells and total cell lysates were subjected to Western blot for cleaved caspase-3 (p17 specific) and caspase-8. **(E)** Bands corresponding to each protein were quantified, and the intensities of each protein were normalized to the intensity of tubulin. **(F)** Quantification of intracellular survival of *M. bovis* in Raw 264.7 cells pretreated for 3 h with BX-795 (10 μM). Cells were harvested at 24 h post infection with *M. bovis* and bacteria number was determined by CFU counting. Data are representative of at least three independent experiments, each performed in triplicate with similar results. The asterisks indicate significant differences compared with untreated cells (^*^P < 0.05, ^**^P < 0.01, ^***^P < 0.001).

It is well-known that IRF3 plays a critical role in innate immunity by regulating inflammation and type-I interferons (IFNs) (Opitz et al., 2006). Recent studies have identified that activated IRF3 binds cytosolic Bax, which results in Bax translocation to the mitochondria and initiation of the intrinsic apoptotic pathway in response to several types of stimuli such as viruses and alcohol (Chattopadhyay et al., 2010; Petrasek et al., 2013). To verify if this pathway was conserved in *M. bovis* infection, we first assessed mitochondrial function by JC-1 mitochondrial transmembrane potential (ΔΨm) assay (Figure 6A). After infection with *M. bovis* for 24 h, green fluorescence (JC-1 monomer form) increased and red fluorescence (JC-1 aggregates form) decreased, indicating low ΔΨm values and thus mitochondrial dysfunction compared against the negative control. Then we further tested the translocation of Bax and cytochrome c. We found that Bax was detected in isolated mitochondria and was reduced in the cytoplasm. In contrast, cytochrome c was released from mitochondria to the cytoplasm after *M. bovis* infection for 24 h (Figure 6B). To investigate if this process was associated with the phosphorylation of IRF3, Raw 264.7 cells were infected with *M. bovis* after pre-treatment with BX-795. The translocation of Bax and the release of cytochrome c were significantly inhibited. Furthermore, we detected the interaction between IRF3 and Bax by co-immunoprecipitation. As shown in Figure 6C, the phosphorylated IRF3 bound to Bax during *M. bovis* infection. Collectively, these data demonstrate that *M. bovis* stimulated the phosphorylation of IRF3 in the cytoplasm, where IRF3 together with bax translocate into the mitochondria to activate mitochondrial outer membrane permeabilization (MOMP), leading to the release of cytochrome c subsequently damaging the organelle and cause apoptotic cell death.

### IRF3 also causes caspase-8 activation and controls intracellular survival of *M. bovis*

Previous studies indicate that the extrinsic cell death regulators FADD and caspase-8 are linked to IRF3 activation in hepatocyte apoptosis (Petrasek et al., 2013; Liu et al., 2015). However, this has not been studied in mycobacterial infection. Raw 264.7 cells treated with BX-795 before *M. bovis* infection were studied on this effect. Caspase-8 and caspase-3 were decreased in a dose-dependent manner in response to BX-795 at 24 h pi (Figures 6D,E). Intracellular survival of *M. bovis* was sustained when BX-795 was used (Figure 6F). These findings show that the extrinsic cell death pathway may also be involved in IRF3-mediated apoptosis. And IRF3 can partly control intracellular survival of bacteria.

**Figure 7:**
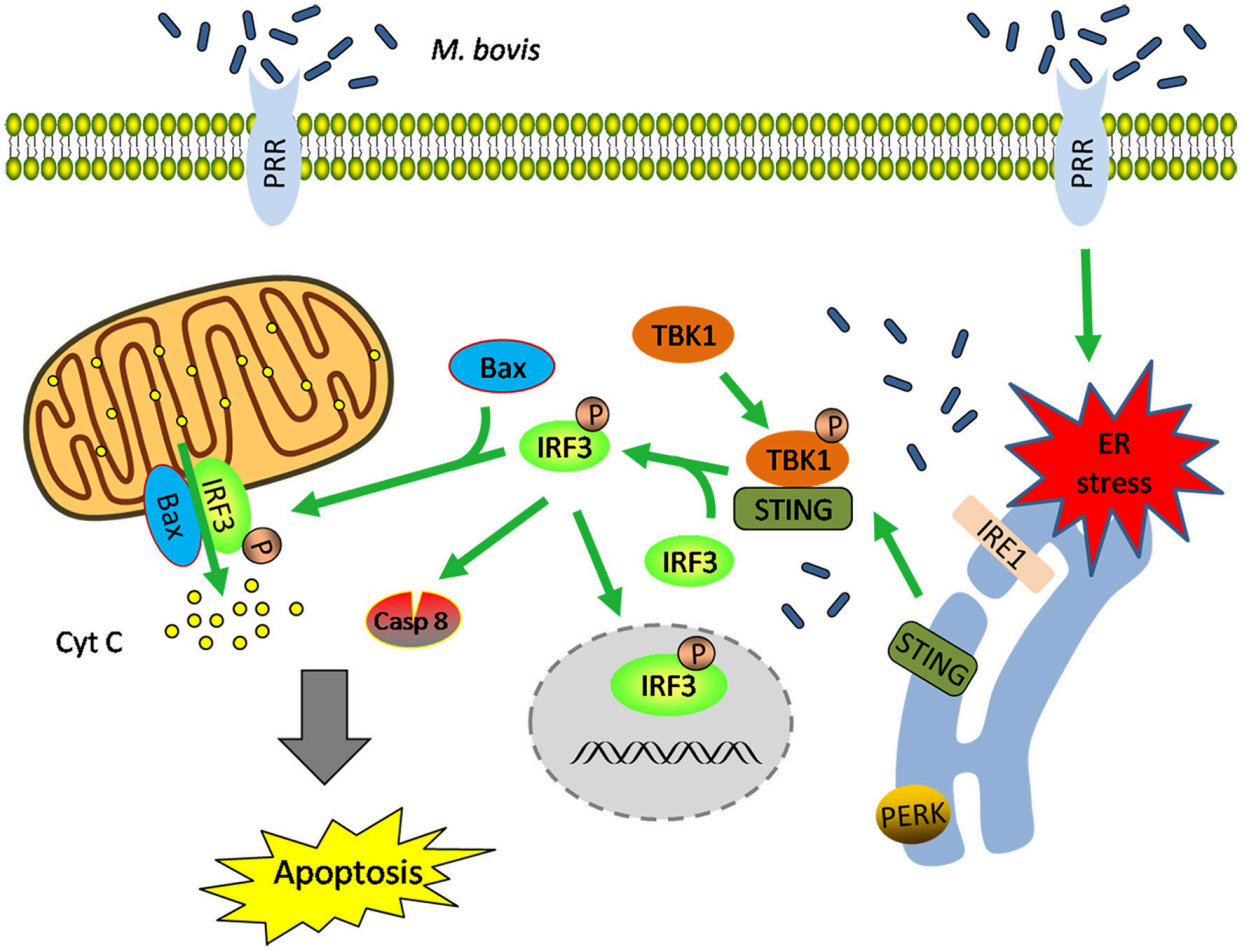
**Schematic representation of ER stress-mediated apoptosis via the activation of IRF3 following ***M. bovis*** stimulation**. *M. bovis* induced ER stress in macrophages triggers the translocation of STING, which facilitates phosphorylation of IRF3 by the downstream phosphorylated TBK1. Subsequently, (i) phosphorylated IRF3 translocates to the nucleus to induce the transcription of putative apoptosis-related factors. (ii) phosphorylated IRF3 associates with Bax and triggers mitochondrial pathway of macrophages apoptosis. (iii) phosphorylated IRF3 activates the downstream caspase-8. All these three pathways finally lead to apoptosis.

These data highlight that (i) *M. bovis*-induced apoptosis is mediated by ER stress and (ii) STING-TBK1-IRF3 pathway is activated by ER stress, which finally leads to apoptosis (Figure 7).

## Discussion

Mycobacteria are highly adapted to macrophages and have multiple mechanisms to resist the host immune responses (Liu et al., 2015). Apoptosis is considered as a crucial outcome for the host to control intracellular bacteria (Aguilo et al., 2013; Divangahi et al., 2013). Thus, it is necessary to understand the exact mechanisms of apoptosis regulation during mycobacterial infection.

Previous studies have identified that a low MOI infection of virulent Mtb leads to macrophage apoptosis, while a high MOI infection induces necrosis (Lee et al., 2006, 2009; Lim et al., 2011). In preliminary experiments, we found that Raw 264.7 cells infected with *M. bovis* at an MOI of 10 induced more apoptosis than at MOI of 1, 5 with few cells showing signs of necrosis. Thus, an MOI of 10 was chosen for these studies. This difference in effective infective dose is likely to be defined by the infecting strain genotype.

In the present study, apoptosis was verified to be caspase dependent. Caspase-3, caspase-9, and caspase-12 were activated by *M. bovis* infection. Caspase-12 is localized to the ER membrane and can be activated by the calcium activated protease calpain, or separating from TRAF2 or the translocation of caspase-7 upon ER stress. Activated caspase-12 translocates to the cytosol, where it directly cleaves pro-caspase-9 that next cleaves caspase-3. This cascade is not dependent on either mitochondria or death receptor pathway (Nakagawa and Yuan, 2000; Yoneda et al., 2001; Szegezdi et al., 2003).

Recent studies suggested that *M. bovis* infection results in loss of Ca^2+^ from the ER and an increase in the intracellular redox state which results in accumulation of unfolded or misfolded proteins in the ER resulting in ER stress (Choi et al., 2010; Lim et al., 2015). In the present study, we first observed the morphology of ER by TEM in order to determine whether ER stress was involved in *M. bovis* infection. The expansion of the ER compartment in early post infection time points prompted us to continue to test the ER stress-related indicators. The ratio of the splicing of XBP-1 mRNA was increased in a time-dependent manner at 9 h pi and gradually decreased by 48 h pi. The phosphorylation of eIF2α was increased in a time-dependent manner in 24 h. These results indicate that the phosphorylated IRE1α mainly activate the downstream transcription factor XBP-1 in early post infection. Then XBP-1s likely binds to the UPR element (UPRE) and to the ERS-response elements I and II (ERSE-I and ERSE-II) in the promoter regions of target genes inducing the expression of a large number of other related genes to reduce or abrogate ER stress and restore ER homeostasis (Cao and Kaufman, 2012). Unlike the protective role of IRE1α pathway in the early stages of infection, the PERK pathway plays an apoptotic role in the late stages. Similarly, an early onset of XBP-1 splicing and a slow translation of ATF4 for cell survival were observed by imaging the single cell response to ER stress (Walter et al., 2015). We also confirmed this apoptosis caused by ER stress is beneficial in controlling the number of intracellular bacteria by pre-treatment with the specific agonist and inhibitor of ER stress.

Emerging evidence suggests that IRF3, a transcription factor regulating innate immune responses, was involved in ER stress-mediated apoptosis (Liu et al., 2012; Petrasek et al., 2013; Jin et al., 2015). However, no reports have shown that this participation of IRF3 upon ER stress links to mycobacterial infections. In general, inactivated IRF3 exists in the cytoplasm. Once ER stress occurs, IRF3 will autophosphorylate and translocate to the nucleus to induce transcription of IFN-β, inflammatory cytokines and apoptosis-related factors (O'Connell et al., 2004; Liu et al., 2012). Furthermore, we detected the phosphorylation and translocation of IRF3 in 24 h. We then confirmed the activation of IRF3 was mediated by ER stress by the pre-treatment with 4-PBA. Next, the activation mechanisms of IRF3 during *M. bovis* infection needed to be clarified. One recent study indicated that different ER stressors use distinct mechanisms to activate IRF3, such as Tm is related to the activation of ATF6 processing, while thapsigargin is linked to the calcium mobilization and the requirement of STING and TBK1 (Liu et al., 2012). Another study has shown that cytosolic dsDNA released from mycobacteria via the ESX-1 secretion system could cause the innate immune response by cGAS-STING pathway (Collins et al., 2015). And also mycobacterial infection could result in loss of Ca^2+^ from the ER (Choi et al., 2010; Lim et al., 2015). Thus, we focused on and hypothesized that the mechanism of IRF3 activation is related to the STING-TBK1 pathway upon ER stress during *M. bovis* infection. STING is an ER-resident protein, harboring three functional domains: A cytoplasmic C-terminal tail, a central globular domain, and four putative N-terminal transmembrane motifs which anchor STING to the ER (Ishikawa and Barber, 2008; Sun et al., 2009). STING is not only highly expressed in immune-related cells, but also plays an important role in function as its evolutionary conservation, such as human STING and mouse STING share approximate 68% sequence identity and 81% similarity at the amino acid level, especially at the C-terminal region (Zhong et al., 2008; Sun et al., 2009). STING undergoes phosphorylation, ubiquitination and translocates to the cytoplasm upon ER stress, which serves as a platform for the recruitment and auto-phosphorylation of the serine-threonine kinase TBK1 and subsequently phosphorylates and activates the transcription factor IRF3 (Liu et al., 2012). In our studies, the phosphorylation of TBK1 and the co-localization of STING and TBK1 were both observed after *M. bovis* stimulation. STING and TBK1 pathway activation was associated with ER stress by the inhibitor 4-PBA. We discovered that STING-TBK1-IRF3 pathway can be activated by ER stress during *M. bovis* infection, but which specific pathway of ER stress mediated the activation of STING-TBK1-IRF3 pathway remains to be determined.

Finally, we investigated how IRF3 induced apoptosis upon ER stress during *M. bovis* infection. Numerous studies have addressed three major mechanisms of IRF3-mediated apoptosis: (i) homodimers of IRF3 induce transcription of the BH3-only proteins Noxa and Puma, which antagonizes pro-survival Bcl-2 proteins such as Mcl-1 and Bcl-xL to activate Bax and Bak and to damage mitochondria, leading to the release of cytochrome c which causes apoptosis. (ii) activated-IRF3 binds cytosolic Bax through a BH3-like domain to the mitochondria to induce the release of cytochrome c which causes apoptosis. (iii) the recruitment of FADD and caspase-8 results in caspase-8 activation, which cleaves Bid to antagonize pro-survival Bcl-2 family members allowing Bax and Bak to induce cell death (Vince and Tschopp, 2010). In the present study, we found the translocation of Bax and the release of cytochrome c. The interaction of IRF3 and Bax was observed by co-immunoprecipitation. Splicing of caspase-8 and caspase-3 was decreased in a dose-dependent manner in response to BX-795. Previous studies show that TBK1 participates in autophagy to control mycobacterium survival (Pilli et al., 2012). But our results document that apoptosis mediated by IRF3 partly control intracellular bacteria which is also in contrast to the results presented in a previous study that showed IRF3^−/−^ mice are better at controlling mycobacteria than wild type mice (Manzanillo et al., 2012). It is likely that our cell models provide a more simplistic albeit pristine look at macrophage-mycobacterium interactions compared to a genetically modified mouse model. Alternately, since our study looked at *M. bovis* vs. *Mtb* by Manzanillo et al., pathogen specific responses, that may explain the differences in findings, cannot be ruled out. Taken together, our results identify a new and essential role of STING-TBK1-IRF3 pathway mediating a cross-talk between ER stress and apoptosis during *M. bovis* infection. To our knowledge, this is the first study to demonstrate a relationship between the activation of IRF3 and apoptosis in *M. bovis* infection. The data indicates that not only ER stress, but also IRF3 induced by ER stress can effectively control intracellular bacteria, which affords an alternate approach for the treatment of TB.

## Author contributions

YC and XZ designed the study; YC performed experiments; YC, CL, WY, ZS, SS, and PB analyzed the data; YC drafted the manuscript; DZ, LY, and XZ contributed to conduct of the laboratory work and critical review of the manuscript. All authors contributed to read and approved the final version.

### Conflict of interest statement

The authors declare that the research was conducted in the absence of any commercial or financial relationships that could be construed as a potential conflict of interest.
